# Cognitive decline limits compensatory resource allocation within the aged swallowing network

**DOI:** 10.1007/s11357-025-01649-y

**Published:** 2025-04-09

**Authors:** Sonja Suntrup-Krueger, Paul Muhle, Janna Slavik, Jonas von Itter, Andreas Wollbrink, Rainer Wirth, Tobias Warnecke, Rainer Dziewas, Joachim Gross, Sven G. Meuth, Bendix Labeit

**Affiliations:** 1 Department of Neurology, University of Münster, Albert-Schweitzer-Campus 1, Building A1, 48149 Münster, Germany; 2 Institute for Biomagnetism and Biosignalanalysis, University of Münster, Münster, Germany; 3 Department of Geriatric Medicine, Marien-Hospital Herne, Herne, Germany; 4 Department of Neurology and Neurorehabilitation, Klinikum Osnabrück, Osnabrück, Germany; 5 Department of Neurology, Medical Faculty, University Hospital Düsseldorf, Düsseldorf, Germany

**Keywords:** Presbyphagia, Swallowing, Cognitive decline, Dual-task, Older people, Magnetoencephalography

## Abstract

Cognitive decline has been postulated to predispose to presbyphagia but the neurophysiological basis of this interaction is unclear. To investigate the role of cognition for compensatory resource allocation within the swallowing network and behavioral swallowing performance in dual-task cognitive and motor interference in ageing, volunteers ≥ 70 years of age without preexisting diseases causing dysphagia were investigated using Flexible Endoscopic Evaluation of Swallowing (FEES) including a cognitive and motor dual-task paradigm and a Montreal Cognitive Assessment. The neural correlates of swallowing during dual-task were characterized using magnetoencephalography. Results were related to cognitive function. Sixty-three participants (77.7 ± 6.1 years) underwent FEES, of which 40 additionally underwent MEG. Both cognitive and motor dual-tasks interfered with swallowing function resulting in an increase in pharyngeal residue and premature bolus spillage. The extent of swallowing deterioration (“dual-task cost”) was associated with cognitive decline (cognitive dual-task: Spearman’s rho =  − 0.39, *p* = 0.002; motor dual-task: Spearman’s rho =  − 0.25, *p* = 0.046). When challenged with dual-tasking participants with regular cognition showed compensatory stronger and broader brain activation in cortical pre- and supplementary motor planning areas as well as in frontal executive regions within the cortical swallowing network (*p* = 0.004) compared to participants with cognitive deficits. They also performed better in the competing cognitive and motor dual-task and showed fewer incorrect responses (*p* = 0.028). Oropharyngeal swallowing involves cognitive cortical processing. Cognitive decline seems to limit the capacity for compensatory resource allocation within the swallowing network. This may lead to deterioration in both swallowing function and concurrent cognitive-motor performance in challenging dual-task situations.

## Background

Oropharyngeal dysphagia is a geriatric syndrome that is highly prevalent in geriatric patients but also community dwelling senior citizens [1–3]. Dysphagia not only limits quality of life but frequently leads to serious complications such as malnutrition [4, 5], aspiration pneumonia [4, 5], and difficulties with medication adherence [6], which places a significant financial burden on the healthcare system [7] and increases mortality [4, 5]. Changes in swallowing function may not only be a consequence of disease, but also a result of (physiological) ageing, which is referred to as primary presbyphagia [8–10]. In the presence of predisposing diseases, age-related changes in swallowing function may limit functional reserve, resulting in manifest dysphagia, also known as secondary presbyphagia [10].

The pathophysiological mechanisms of age-related swallowing impairment are poorly understood and assumed to be multifactorial [10]. Pharyngeal hypesthesia seems to be an important factor [3, 11, 12]. In addition, sarcopenia is considered a relevant mechanism for decreased pharyngeal contractility [13–15]. Moreover, the central control of swallowing has increasingly come into focus. In contrast to historical models in which swallowing was conceptualized as a brainstem reflex, neuroimaging studies have repeatedly shown that a complex cortical network is involved in swallowing coordination [16–22]. Consistent with this, behavioral studies suggest that cognitive and attention-driven processing is essential in swallowing control. Thus, studies in healthy participants and Parkinson’s disease (PD) have shown that oropharyngeal swallowing is altered when distracted by a second attention-demanding task (dual-task) [23–32]. Further, studies in different geriatric patient groups have shown an association of oropharyngeal dysphagia with cognitive impairment, although the data are partly inconclusive [33].

Neural correlates of compensation strategies for presbyphagia and their relation to cognitive function have not yet been investigated in detail. Our objectives were as follows: (1) to characterize dual-task interference with presbyphagia in older age, (2) to investigate the association of dual-task interference with cognitive impairment, and (3) to delineate neural correlates of swallowing during dual-task in relation to cognitive status. Community-dwelling older volunteers underwent a cognitive assessment, Flexible Endoscopic Evaluation of Swallowing (FEES) and magnetoencephalography (MEG), during a dual-task paradigm.

## Methods

### Participants

This study was part of a larger project focussing on clinical determinants and neural correlates of presbyphagia [3]. Community-dwelling individuals aged ≥ 70 years were recruited via newspaper advertisements and flyers or presentations at senior meetings. Only individuals without preexisting diagnosis associated with dysphagia were included. A screening for cognitive impairment was performed using the Montreal Cognitive Assessment (MoCA) [34].

### Endoscopic swallowing dual-task protocol

Presbyphagia was assessed with FEES, which, along with videofluoroscopy, is considered the diagnostic gold standard for oropharyngeal dysphagia [35]. A FEES dual-task protocol was conducted as described in detail elsewhere [23, 24]. In brief, three sets of tasks were performed: A baseline FEES without dual-task interference followed by a FEES during a cognitive and a motor dual-task. In each of the task-sets, three different consistencies were tested in the following order: three trials of 8 ml of green jelly (semisolid), three trials of 5 ml blue-dyed liquid, and three trials of white bread (solid) with a size of approximately 3 × 3 × 0.5 cm. During the cognitive dual-task, participants were instructed to repeat a six-digit number continuously in mind. During the motor-dual-task, participants were given clicking devices (AFUNTA, Mini Hand Tally Counter) in each hand and instructed to click alternately left and right as quickly as possible.

### Rating of swallowing function

The following items were assessed for each swallowing trial: (1) premature bolus spillage, (2) penetration and aspiration, and (3) residue in the pharynx. A previously established swallowing-score validated to detect clinically relevant changes in swallowing function was applied [36]. Each of the swallowing pathologies is rated on a scale from 0 (normal) to 4 (severe impairment) for every trial and each food consistency, contributing to an overall cumulative swallow score ranging from 0 to 108. The “dual-task cost” was calculated by dividing the swallow score in the respective dual-task condition by the swallow score at baseline.

### MEG study and analysis protocol

MEG was used as neuroimaging modality to compare swallowing network activation during a swallowing dual-task paradigm in participants without cognitive impairment (MoCA ≥ 26) and participants with cognitive decline (the participants with the lowest MoCA score in the cohort). Magnetoencephalography (MEG) detects local power changes in oscillatory cortex activity evoked by a decrease or increase in synchrony of generating neuronal populations. Voluntary movements are generally accompanied by a power decrease (event-related desynchronization, ERD) during the task, followed by a power increase (event-related synchronization ERS) after movement execution. There is general agreement that ERD reflects cortical activation or arousal [37]. The MEG measurements were performed with a 275-channel whole-head device (Omega2005 WC, CTF Systems Inc., Canada). Well-established recording parameters were applied, as previously described in detail elsewhere [20]. Water was continuously supplied via a tube in the mouth to facilitate regular swallowing during the MEG examination. Submental EMG electrodes were used to record the activity of the suprahyoid swallowing muscles to study event-related brain activation. EMG power (root mean square (RMS) value) and peak-to-peak amplitude were assessed. In addition to self-paced swallowing, participants had to perform a combined cognitive and motor task. They were instructed to watch a screen on which a square, a triangle, or an octagon was presented in randomized order. Each of the symbols was shown for 500 ms with a jittered stimulus-onset interval of 3000 ± 500 ms (Stimulus control software: Presentation, Neurobehavioral Systems Inc., USA). Participants were instructed to press a button either with the left or right index finger, or to rest, depending on the symbol. Reaction time and correctness of responses were analyzed. Head movement was monitored to ensure data quality. Number of swallows during the measurement was counted to guarantee correct task performance.

MEG data analysis followed a standard pipeline established by our group and applied in manifold studies on the cortical control of swallowing [19, 20, 22]. In summary, the MATLAB-based (MathWorks Inc., USA) software toolbox “FieldTrip” (http://www.ru.nl/fcdonders/fieldtrip) was used [38]. Swallowing acts were identified according to coregistered EMG data. MEG raw data was filtered within theta (4–8 Hz), alpha (8–13 Hz), beta (13–30 Hz), low (30–60 Hz), and high gamma (60–80 Hz) frequency ranges. Source localization of each participant’s task-related cortical activation changes was performed with a linearly constrained minimum variance beamformer technique. After that, three-dimensional source localization maps were spatially normalized to a template MNI brain using SPM8 (http://www.fil.ion.ucl.ac.uk/spm) to compute grand averages within groups. To identify significant brain activation differences between groups with vs. without cognitive impairment, data were compared applying a cluster-based nonparametric randomization approach built into FieldTrip.

### Statistical analysis

Descriptive statistics were used for demographics and swallowing function. The Friedman Test followed by post-hoc comparison was used to compare FEES swallowing scores during the baseline, cognitive, and motor dual-task conditions. Spearman’s rho was used to analyze the correlation between MoCA score and dual-task cost.

For MEG participants’ group comparison, the independent sample *t*-test was applied on metric data (age, swallow count in MEG) and the Mann–Whitney *U* test for nonparametric data (MoCA, swallowing scores and dual-task cost, head movement in MEG, EMG parameters, response latency, and correctness in MEG dual-task condition). For nominal data (sex), the chi-square-test was used.

## Results

### Participant cohort and baseline FEES

Of the 64 participants participating in the previous study part [3], 63 also underwent the dual-task swallow examination in FEES. Characteristics of the participant cohort can be found in Table 1. Approximately half of all participants showed mild presbyphagia with pharyngeal residue and/or premature bolus spillage, whereas penetration and aspiration occurred rarely.

**Table 1:** Demographic, clinical, and swallowing physiological data of the participant cohort

Parameter	Value
Demographics	
Mean age in years ± SD	77.7 ± 6.1
Men/women, n (%)	25 (39.7%)/38 (60.3%)
Cognition	
Median MoCA (mode, range)	25 (24, 18–30)
Swallowing parameter	
No presbyphagia, n (%)	32 (50.8%)
Mild presbyphagia, n (%)	29 (46.0%)
Moderate presbyphagia, n (%)	2 (3.2%)
Severe presbyphagia, n (%)	0 (0%)
Median total swallowing score (mode, range)	12 (12, 1–37)
Median spillage swallowing score (mode, range)	6 (3, 0–17)
Median penetration/aspiration swallowing score (mode, range)	0 (0, 0–5)
Median residue swallowing score (mode, range)	5 (4, 0–25)

*SD* standard deviation; *MoCA* Montreal Cognitive Assessment

### Comparison of FEES dual-task conditions

The total swallowing score [*χ*2(2) = 42.7, *p* < 0.001] as well as the sub-scores for premature bolus spillage [*χ*2(2) = 48.5, *p* < 0.001] and pharyngeal residue [*χ*2(2) = 13.3, *p* < 0.001] differed between task conditions. For penetration and aspiration, however, there were no differences between these conditions [*χ*2(2) = 0.1, *p* = 0.94]. The post hoc test revealed that oropharyngeal swallowing worsened in both the cognitive and motor dual-task compared to baseline. This deterioration arised from increases in pharyngeal residue and premature bolus spillage (Table 2).

**Table 2:**
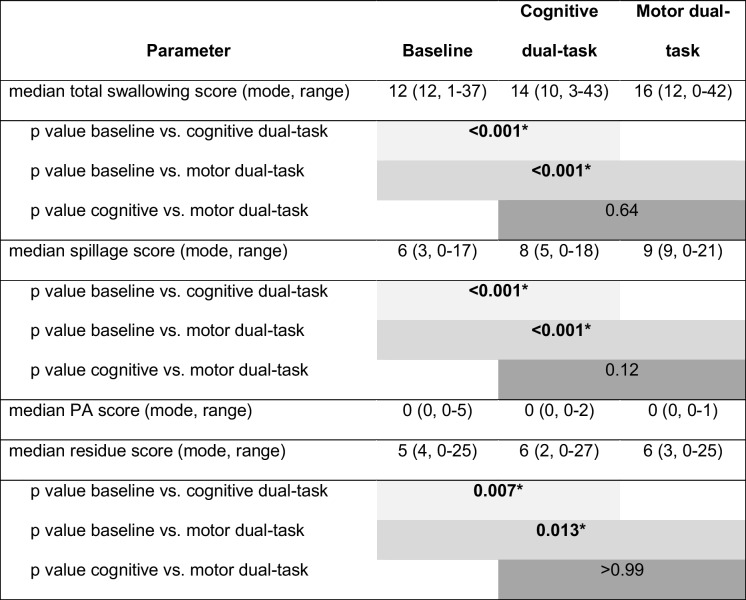
Comparison of the swallowing parameters during baseline and the dual-task conditions

*PA* penetration and aspiration

^*^Significant *p*-values

### Association of FEES dual-task cost with cognition

The MoCA score was inversely correlated with both cognitive (Spearman’s rho =  − 0.39, *p* = 0.002) and motor dual-task cost (Spearman’s rho =  − 0.25, *p* = 0.046) suggesting an association of cognitive decline and swallowing deterioration in both conditions.

### MEG cohort

Of 63 participants, 47 also completed the MEG swallowing dual-task paradigm (4 participants aborted the investigation; in 5 participants, technical issues prevented recording; and in 7 participants, EMG recording was insufficient). Twenty participants had a MoCA score above the generally accepted cut-off of ≥ 26. Due to the requirements for statistical MEG data analysis, an equal-sized comparison group including the 20 participants with the lowest MoCA score was built. Demographic, clinical, and FEES parameters of these groups can be found in Table 3. According to group categorization, there was a significant difference in the MoCA score. Furthermore, there was a tendency towards older participants and higher cognitive dual-task costs in the group with decreased cognition. Swallowing function at baseline without distraction was comparable.

**Table 3:** Comparison of demographic, clinical, and swallowing parameters, as well as MEG task performance between the MEG-participants with normal and decreased cognition

Parameter	MoCA ≥ 26	MoCA ≤ 24	*p* value
Mean age ± SD	75.5 ± 4.5	79.0 ± 6.4	0.05
Sex m/f	6/14	11/9	0.11
Median MoCA (mode, range)	28 (27, 26–30)	23 (23, 18–24)	< 0.001*
FEES			
Median total swallowing score (mode, range)	13 (12, 5–37)	12.5 (12, 2–35)	0.37
Median cognitive dual-task cost (range)	1.1 (0.4–1.9)	1.3 (0.6–5.0)	0.07
Median motor dual-task cost (range)	1.2 (0.7–1.8)	1.3 (0–3.0)	0.22
MEG			
Max. head movement (mm) (mean ± SD)	4.5 ± 2.1	7.0 ± 6.9	0.42
No. of swallows ± SD	42 ± 12	35 ± 13	0.10
EMG power (µV) (mean ± SD)	115.6 ± 145.9	127.2 ± 196.5	0.84
EMP peak-to-peak amplitude (µV) (mean ± SD)	414.4 ± 269.9	452.5 ± 338.0	0.57
Incorrect/missing responses, left (*n* ± SD)	0.5 ± 0.8	2.0 ± 2.2	0.028*
Reaction time for button pressing, left (ms ± SD)	571.3 ± 59.5	631.2 ± 139.4	0.09
Incorrect/missing responses, right (*n* ± SD)	0.9 ± 1.3	1.9 ± 2.2	0.06
Reaction time for button pressing, right (ms ± SD)	560.8 ± 54.0	639.1 ± 130.2	0.10

*EMG* electromyography, *f* female, *m* male, *MoCA* Montreal Cognitive Assessment

^*^Significant *p*-values

### MEG results

Performance in the MEG dual-task condition is also shown in Table 3. Head movement, swallow count, and EMG parameters were comparable between groups, ensuring all participants swallowed regularly. Participants with lower MoCA had significantly more incorrect or missing responses in the interfering button-press task especially when required to press the left-hand button.

**Fig. 1:**
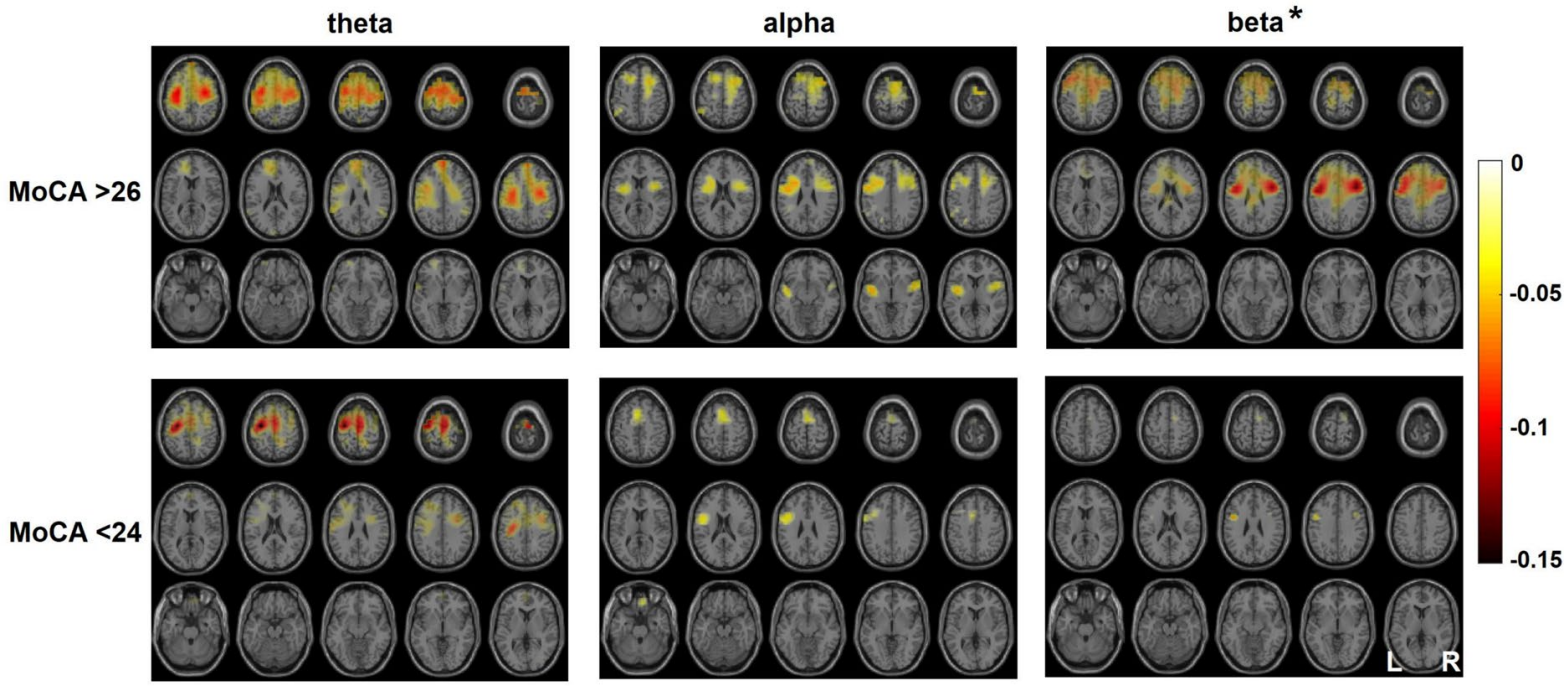
Grand average of swallowing-related brain activation per group in participants with regular (top row) vs. impaired cognition (bottom row). Anatomical localization and color-coded intensity of event-related desynchronization (ERD) of oscillatory brain activity during swallowing is displayed for separate frequency ranges (theta =  4-8 Hz, alpha = 8–13 Hz, beta = 13–30 Hz). Negative intensity values (0 up to − 0.15) denote ERD of brain oscillations in comparison to baseline resting state; asterisk (*) indicates statistically significant difference between participant groups

After calculating grand averages of event-related desynchronization of oscillatory brain activity during swallowing within each group, task-related changes compared to resting baseline were observed in the theta, alpha and beta frequency range (for detailed information, see Table 4 and Fig. 1). Activation was generally more widespread in participants with good cognition, involving not only the core sensorimotor swallowing areas but especially extending into the frontal lobe. Statistical comparison revealed activation differences to be significant in beta (*p*-value 0.004, see Fig. 1). Participants with good cognition had stronger activation in the pre- and supplementary motor planning areas (PMC, SMA, Brodmann Area, (BA) 6). Activation was also increased in parts of the frontal lobe including the orbitofrontal, rostrolateral, dorsolateral (DLPFC), and medial prefrontal cortex (BA 8–11). Also, the supramarginal gyrus (BA 40) of the inferior parietal lobe (IPL) and middle temporal gyrus (BA 21) demonstrated significantly stronger activation.

**Table 4:** Peak location and intensity of swallowing-related changes in brain activation per group. Negative values denote event-related desynchronization of oscillatory brain activity in comparison to baseline

Parameter	Peak value	MNI coordinates	Brain area
MoCA ≥ 26			
Theta	− 0.106	[− 30, − 12, 49]	Left middle frontal gyrus
Alpha	− 0.068	[− 47, 0, 30]	Left precentral gyrus
Beta	− 0.143	[39, 0, 32]	Right precentral gyrus
MoCA ≤ 24			
Theta	− 0.159	[− 29, − 14, 59]	Left precentral gyrus
Alpha	− 0.055	[− 47, 2, 20]	Left inferior frontal gyrus
Beta	− 0.083	[− 47, 0, 30]	Left precentral gyrus

*MNI* Montreal Neurological Institute, *MoCA* Montreal Cognitive Assessment

## Discussion

The main findings of this study are as follows: (1) Both cognitive and motor dual-tasks led to deterioration of swallowing, presenting as an increase in premature bolus spillage and pharyngeal residue. (2) The extent of deterioration was associated with cognitive decline. (3) Participants with good cognition revealed stronger activation in brain regions involved in motor planning and executive frontal functions during dual-task swallowing. Thereby, they showed better performance in a simultaneous cognitive-motor button-press task.

Thus, the combined behavioral and neuroimaging results suggest that attentional processing and cognition are relevantly involved in the cortical control of swallowing, not only in the oral phase (worsening of premature spillage) but also in the pharyngeal phase (increase of residue). In contrast to historical assumptions in which pharyngeal swallowing was considered a mere brainstem reflex, swallowing appears to be substantially influenced by cortical cognitive input [39]. The dual-task paradigm used in this study seems to have resulted in depletion of cortical reserve capacity, especially in the group of participants with cognitive deficits. This led to deterioration of oropharyngeal swallowing function associated with cognitive decline and also to a worse performance in the parallel cognitive-motor button-press task, indicating that exploited cortical processing capacities limit performance of both actions in a dual-task situation. In contrast, in the group of participants without cognitive deficits, cortical cognitive processing was (at least partially) maintained during dual-task swallowing, as evidenced by stronger brain activation and less dual-task costs. The interpretation that the ability to recruit additional processing resources for effective swallowing in challenging situations depends on cognitive capacity is further supported by a closer look into the MEG data. Both groups demonstrated a similar frequency range, in which task-related changes were observed: weakest activation was found in alpha, which has specifically been linked to somatosensory processing, indicating this was not the main issue in dual-task swallowing. Theta oscillations were relatively prominent in premotor and frontal areas. Theta is a feature of attentional processes and known to relate to the overall demands of a task [40] and to the initiation of self-directed movement [41]. Significant differences were observed in beta. Event-related desynchronization (ERD) of the beta rhythm constitutes a correlate of an activated cortical area [42]. Enhanced beta activity, as found in the group with good cognitive function in premotor an frontal lobe areas, has been hypothesized to imply that the cortex is “primed” by motor preparation or focussed attention, the latter being essential in complex dual-task situations.

Apart from swallowing, there is substantial literature interpreting age-related increases in neural recruitment as a compensatory mechanism that varies as a function of task complexity and the availability of additional neural resources [43]. Processing inefficiencies cause the aging brain to recruit more neural resources to achieve computational output equivalent to that of a younger brain [44]. Since we have no young control group in our study but compare older people with good vs. reduced cognition, the application of these theories to our data has to be made with caution. The posterior-anterior shift in aging (PASA) is a model describing age-related reduction in occipital activity coupled with compensatorily increased frontal activity [45]. Prefrontal regions show the greatest evidence for age-related atrophy, and yet, paradoxically, these are the sites where overactivation and evidence for compensation tend to be most pronounced [44]. While we indeed observed increased prefrontal activation in the demanding dual task situation in line with the PASA hypothesis, we cannot comment on occipital activation as this is typically not observed during motor swallowing. Second, we only observed this additional recruitment in older participants with good MoCA. This may be explained by the CRUNCH theory [44]. The Compensation-Related Utilization of Neural Circuits hypothesis proposes that as task difficulty increases, more brain areas will get involved. Older adults reach their load capacity sooner than younger ones, so at easy and intermediate levels of task difficulty, they will compensate effectively by recruiting more neural resources than younger adults. At higher levels of load, when overcoming their individual “CRUNCH point” [44], the compensatory mechanism is no longer effective, leading to less activation and poorer performance in older adults. The activation pattern in participants with good cognition was similar to what has been described during self-paced swallowing in older participants without divided attention [3]. This indicates sufficient “cortical reserve” to keep the swallowing network stable even when challenged with a higher processing load. Consistent with the CRUNCH theory, our participants with cognitive decline showed less activation and worse performance, maybe because their individual level of load was so high as to overcome their individual “CRUNCH point.” The premotor cortex and supplementary motor area are considered to be involved in planning, initiating, and executing complex coordinated movements [46]. Thus, stronger beta activation in these areas in the group with normal MoCA seems a compensation attempt to sustain effective task performance in motor challenges. This finding is supported by previous MEG studies indicating spectrally specific age-related changes of movement-related oscillatory signatures especially in beta and gamma, reflecting the cognitive components of a task [43]. Arif et al. [47] recently observed stronger beta oscillations with increasing age by cognitive interference of a motor task in the right dorsolateral prefrontal cortices, left parietal, and medial parietal regions. In line with that, activation differences were prominent in the DLPFC, rostrolateral prefrontal cortex, and IPL in our study. The anterior prefrontal cortex is thought to be engaged in the simultaneous execution of multiple tasks. “Cognitive branching,” i.e., to keep a running task in a pending state for subsequent execution upon completion of another one, is a core function [48]. Adding to this, the DLPFC, together with the PMC and IPL, constitutes an interface between attentional control and sensorimotor integration, which was found to be altered in patients with functional dysphagia [49]. For swallowing, the IPL is thought to process afferent information from the oropharynx and to integrate it with ongoing motor output [50]. In two recent fMRI studies comparing swallowing in young vs. older volunteers, bilaterally increased activation in the rostrolateral prefrontal cortex (BA10) as well as increased pericentral and inferior frontal cortex activation was observed in senior participants [16, 18].

Brain areas overlapping with those found active in our study have also been described in other multiple task paradigms unrelated to swallowing. A meta-analysis of neuroimaging studies of dual-task or task-switching revealed a common core network in fronto-parietal regions. In addition, specific activations related to dual-tasking were found in the bilateral frontal operculum, dorsolateral PMC, anterior intraparietal sulcus, left inferior frontal sulcus, and left inferior frontal gyrus [51]. Given the overlap with the areas described in our study, the activation pattern could (partly) also represent a general correlate for dual-task processing. Increased activation in participants without cognitive deficits could then be explained by their generally higher capacity for dual-task processing.

An important limitation to be considered when interpreting our results is that there is no unified theory of how dual-task costs arise [52]. The bottleneck theory explains dual-task costs in the context of limited processing capacity. According to this theory, attention-driven processing requires multiple processing channels, and each channel is limited in capacity. During dual-tasking, one task is assumed to occupy the bottleneck channel, limiting the processing resources available for the other. This results in degraded performance or speed for one or both dual tasks [52]. According to the capacity sharing theory, the limitation of cognitive resources is not seen as a limiting phase of processing, but rather as the result of a common pool of resources shared between multiple tasks [52].

The behavioral results of this study are in line with other motor dual-task interferences. A meta-analysis on gait parameters of older adults demonstrated that adding a dual-task condition significantly reduced gait speed and worsened cadence, stride time, and measures of gait variability [53]. As in our study, an association between dual-task cost and cognitive deficits was observed [54]. Further, dual-task swallowing physiology studies have mostly yielded results consistent with our findings. Even in healthy young participants, swallowing capacity [25] and volume per swallow [27 28] decrease when adding a cognitive or motor dual-task. In another study in 10 healthy participants, however, there was no evidence of dual-task interference between oropharyngeal swallowing time and reaction time to auditory stimuli. In contrast, the anticipatory phase of swallowing, relative to the oropharyngeal phase speeded up in the dual-task condition [29]. A FEES-based study on 27 young participants using the same dual-task paradigm as in this study consistently showed an increase in pharyngeal residue and premature bolus spillage. However, unlike the present data, only motor dual-task interference was observed, whereas the cognitive task did not compromise swallowing [24]. Young participants were probably able to recruit sufficient cognitive reserves to cope with both tasks simultaneously. This observation is also consistent with stronger cognitive dual-task interference in gait in older compared to young participants [54].

Cognitive decline has also been associated with manifestation of dysphagia in a number of neurological diseases, including PD, stroke, and dementia [33]. In a FEES-based study, PD patients showed an increase in pharyngeal residue when performing a thumb-opposition task [31]. Similar to our results, this study also showed an association between cognitive impairment and dual-task cost. In a further study, penetration aspiration scale was assessed in 20 dysphagic PD patients during a cognitive dual-task paradigm using videofluoroscopy [26]. Consistent with the results presented here, patients without as well as those with severe cognitive deficits showed no deterioration in laryngeal penetration or aspiration in the dual-task condition. It is therefore possible that airway protective mechanisms, unlike pharyngeal residue and premature bolus spillage, rely more on reflexive rather than cognitive cortical processing, making it less susceptible to dual-task interference.

## Conclusion

In summary, it seems the swallowing network essentially retains its function in old age but older people need to make increased effort and utilize additional attention to uphold sufficient swallowing performance. Cognitive decline seems to limit the ability for this compensation mechanism, which can be unmasked in dual-task situations—neurophysiologically and clinically. Elderly persons with cognitive impairment should avoid distractions during mealtime and focus on eating.
